# Thyrotoxic Periodic Paralysis: Case Reports and an Up-to-Date Review of the Literature

**DOI:** 10.1155/2011/867475

**Published:** 2011-09-22

**Authors:** Abbi Lulsegged, Christina Wlodek, Michela Rossi

**Affiliations:** ^1^Department of Endocrinology, South London HealthCare NHS Trust, Orpington Hospital, Sevenoaks Road, Orpington BR6 9JU, UK; ^2^Department of Endocrinology, Whittington NHS Trust Hospital, London N19 5NF, UK

## Abstract

*Objectives*. To describe 2 cases of thyrotoxic periodic paralysis. *Methods*. We report of 2 cases of thyrotoxic periodic paralysis in 2 individuals from 2 different backgrounds with emphasis on their presentation and treatment. We also conducted a literature search to put together an update review of thyrotoxic periodic paralysis. *Results*. A 47-year-old Chinese and 28-year-old Caucasian male presented with profound yet reversible weakness associated with hypokalemia on admission bloods and thyrotoxicosis. Both were given definitive therapy to prevent recurrence of attacks with any future relapse of thyrotoxicosis. *Conclusion*. Thyrotoxic periodic paralysis (TPP) is a rare but potentially serious complication of thyrotoxicosis resulting in temporary but severe muscle weakness. Recent discovery of a novel mutation in the KCNJ18 gene which codes for an inwardly rectifying potassium channel and is controlled by thyroid hormones may provide greater insight into the pathogenesis of TPP.

## 1. Introduction

Thyrotoxic periodic paralysis (TPP) is the most common cause of acquired hypokalaemic periodic paralysis. This condition is often transient but potentially serious. It is classically associated with thyrotoxicosis and hypokalemia. Prompt recognition of the problem allows for proper short- and long-term management of this condition.

## 2. Case Reports

A 47-year-old Chinese gentleman presented acutely with a one week history of lower limb weakness and a three-hour history of total lower limb paralysis on wakening. He also complained of palpitations over the previous year. He denied any family history of thyroid or neurological problems. The lower limb weakness had resolved by the time he was seen in hospital.

On examination his upper and lower limb power was 5/5 throughout with no fatigable weakness. Cardiovascular, pulmonary, and abdominal examinations were unremarkable. He was clinically euthyoid with no tremor, nail changes, or thyroid eye signs. There was no palpable goitre. His serum potassium on admission was 3.1 mmol/L, free thyroxine (T4) level 38.5 pmol/L (9.4–22.7), free triiodothyronine (T3) level 14.4 pmol/L (3.5–6.5), and TSH < 0.01 mU/L (0.35–5.50). ECG showed sinus tachycardia only. 

He was given carbimazole 40 mg daily, propranolol, potassium iodide, and lithium so as to promote a rapid euthyroid state and thereby allow for a thyroidectomy which he had several weeks later. A thyroidectomy was performed rather than administration of radioactive iodine because it would have been very difficult for the patient to attend for the blood tests and follow-up appointments required after radioactive iodine.

The second case involved a 28-year-old Caucasian gentleman who presented with nocturnal leg weakness, pain in the muscles of both legs and proximal arms. He awoke the morning of his admission with profound leg weakness and could not get out of bed. He had flaccid weakness of the arms and legs and generalised hyperreflexia. Urgent electromyography and nerve conduction studies were normal. His serum potassium was 2.6 mmol/L, TSH < 0.01, fT4 54 pmol/L (12–22) and TSH receptor antibodies were elevated consistent with Graves' disease and thyrotoxic periodic paralysis. He was started on propranolol 20 mg twice daily, carbimazole 40 mg daily and subsequently had radioactive iodine.

## 3. Discussion

Thyrotoxic periodic paralysis (TPP) is one of the causes of hypokalemic paralysis (HP). HP is a condition characterised by muscle weakness associated with changes in potassium levels. HP can occur either due to transient shifts (hypokalemic periodic paralysis or HPP) or reduction in absolute potassium levels (nonhypokaelmic periodic paralysis) [1]. 

Familial cases are inherited in an autosomal dominant manner. Mutations in *CACNA1S* and *SCN4A* gene adversely affect the function of calcium and sodium ion channels servicing muscle cells, respectively [2]. The KCNJ2 gene codes for inward rectifying potassium channels (Kir 2.1) that moves potassium ions into the cells of skeletal and cardiac muscles. Mutations of this gene have been known to cause familial periodic paralysis with arrhythmias and Andersen-Tawil syndrome [3, 4]. 

Young Oriental men are more likely to be affected with a prevalence of around 2% of all thyrotoxic cases compared to 0.2% in thyrotoxic Caucasian patients [5]. However this condition can affect individuals from different parts of the world [6, 7]. 

It can present as a medical emergency. Patients often have an abrupt onset of paralysis which could affect all four limbs traditionally associated with hypokalaemia. Suspected cases of TPP with normal potassium levels during an attack have been reported although it is worth noting that techniques for taking blood may lead to spuriously higher levels of potassium [8]. The classical presentation is of ascending lower limb paralysis in the early hours of the morning, or after rest following strenuous exercise and a high carbohydrate meal, leaving the patient unable to move. Acute episodes may be preceded with muscle aches, cramps, or stiffness. It is rare to observe ocular, bulbar, or respiratory muscle involvement. Tendon reflexes are generally diminished or absent (although the patient in our second case report had hyperreflexia) with intact sensation and consciousness [9].

Attacks are typically transient and last hours to days and may be triggered by a number of conditions. These include carbohydrates—especially refined carbohydrates and after exercise. Other precipitating factors include trauma, exposure to hot weather, upper respiratory tract infections, emotional stress, menses, medications (diuretics, insulin, glucocorticosteroids, and acetazolamide), alcohol, or recreational drugs such as 3,4-Methylenedioxymethamphetamine (ecstasy) [10–13].  Table 1 summarises the important triggers.

The Na/K+/ATPase pump located on the sarcolemma of muscle cells prevents accumulation of intracellular sodium. It pumps out 3 Na+ for every 2 K+ ions it pumps into the cell. The influx of potassium into the cells alters the resting potential of the muscle cell leaving it unable to depolarise because it is hyperpolarised. The pump is activated by catecholamines (via beta-2 receptors) and therefore in susceptible patients, thyroid hormones since they sensitise the circulation to circulating catecholamines. Thyroid hormones can also directly stimulate Na/K/ATPase activity, and thyrotoxicosis (with or without periodic paralysis) has been shown to increase pump numbers [14, 15]. This pump is also activated by androgens, and men are approximately 10–20 times more likely to be affected than women, with onset in the 4-5th decade of life [16]. 

Insulin activates the Na+/K+/ATPase pump causing an influx of potassium into cells and thus anything that promotes hyperinsulinaemia such as high glycaemic and/or refined carbohydrates can trigger or exacerbate the underlying problem. Euglycaemic hyperinsulinaemic clamp studies have shown that patients with thyrotoxic periodic paralysis may be more insulin resistant than age/sex-matched controls and therefore more likely to have hyperinsulinaemia [17]. The combination of hyperinsulinaemia and TPP is associated with increased Na/K/ATPase activity [18].

As always a detailed clinical history and thorough clinical examination may reveal clues. Thyrotoxic features maybe absent or very subtle; therefore the clinician must have a high index of suspicion [19]. 

A spot urine test for electrolytes and potassium excretion can be very useful. Normal acid base status and low urinary potassium levels are characteristic of hypokalaemia in TPP. It is also important to appreciate that serum potassium levels may be normal in between attacks. Transient hypophosphatemia and hypomagnesemia have been documented alongside elevated creatine phosphokinase. These disturbances resolve as the underlying thyrotoxicosis is treated. Furthermore some authors have suggested that measuring the urinary calcium/phosphate ratio may help diagnose this otherwise rare condition since thyrotoxicosis is associated with increased urinary calcium excretion and TPP with hypophosphataemia [20]. 

Thyrotoxic periodic paralysis, as the name suggests, is associated with thyrotoxicosis. Any cause of thyrotoxicosis maybe sufficient to trigger attacks in susceptible patients. Indeed the condition has been reported in one patient with hypothyroidism who was overreplaced with thyroxine and in another who took triiodothyronine (T3) to facilitate weight loss [21, 22]. The serum thyroid-stimulating hormone level is low or suppressed accompanied by raised free thyroxine (fT4) and triiodothyronine (fT3) levels consistent with primary thyrotoxicosis. Rarely some patients may have central thyrotoxicosis in which case the TSH is detectable and associated with elevated fT4 and fT3 levels [23]. Milder forms of thyrotoxicosis have been associated with thyrotoxic periodic paralysis adding to the diagnostic difficulties [24]. 

An electrocardiogram may show characteristic features of hypokalaemia. Unlike hypokalaemia from other causes, sinus tachycardia predominates in TPP patients. Other findings include atrial fibrillation, atrioventricular block, ventricular fibrillation, and asystole [25, 26]. The electrolyte disturbance can be severe enough to cause asystole and cardiac arrest [27]. 

The underling hyperthyroid state has to be addressed in order to definitively rectify the condition; however initially potassium replacement maybe needed to hasten muscle recovery and prevent cardiopulmonary complications [28]. Potassium chloride is the preferred salt as its is less irritant [29]. Supplementation must be given with caution (not faster than 10 mmol/hour) as there is a risk of rebound hyperkalemia once the muscle cells recover releasing potassium and phosphate into the circulation [30]. For this reason we would recommend using potassium supplementation (especially intravenously) for those patients or at risk of cardiopulmonary arrest. 

A nonselective beta blocker such as propanolol can rapidly promote recovery without the risk of rebound hyperkalaemia [31]. Propranolol blunts the hyperadrenergic stimulation of the Na-K-ATPase pump [32]. Patients should be educated to avoid precipitating factors and continue propranolol until a euthyroid state is achieved so as to prevent symptoms.

Antithyroid medication and thyroidectomy or radioiodine therapy are mandatory to secure a complete cure of TPP and prevent recurrence. There has been a case report of radioactive iodine use being associated with early recurrence of TPP—radioactive iodine can be associated with transient thyroiditis/hyperthyroidism [33]. Therefore caution is recommended if radioactive iodine is chosen as the method of delivering definitive treatment and should perhaps be avoided if the patient has or is at risk of cardiovascular disease or had a history of significant arrhthymias.

Phenotypically TPP resembles familial hypokalaemic periodic paralysis, which is a channelopathy. A hypothesis surrounding this similarity recently led to the discovery of mutations of an inwardly rectifying potassium (Kir) channel Kir2.6. It is expressed in skeletal muscle and is transcriptionally regulated by thyroid hormone. The gene KCNJ18 was discovered to code for this Kir2.6 channel which promotes a greater influx of potassium into the cells. Kir2.6 mutations were discovered to be present in 25–33% of unrelated TPP patients in a recent study [34]. Some of these mutations altered a number of Kir2.6 properties and hence muscle membrane excitability leading to paralysis. Kir channelopathies contribute to a number of human diseases including episodic muscle and developmental features of Anderson-Tawil syndrome (Kir2.1), the renal tubular secretion defects of Bartter syndrome type III (Kir1.1), and defective insulin secretion in persistent hyperinsulinemic hyperglycemia of infancy (Kir6.2). 

Through electrophysiological tests, Ryan et al. revealed that some of the Kir2.6 mutations have large effects on currents either inherently or via thyroid hormone inducible mechanisms, such that these mutations may cause predisposition for episodic weakness seen only during thyrotoxicosis [34]. This genetic test is not yet carried out in the UK, therefore our patient was not tested for a mutation of Kir2.6. 

In summary this recent finding has affirmed the complex and widespread role of thyroid hormones in metabolic function and more specifically in TPP. It has also uncovered at least one type of genetic predisposition for the condition. This channelopathy confirms the importance of considering TPP in a patient with sudden onset weakness or paralysis associated with hypokalaemia and should make one specifically search for signs and symptoms of thyrotoxicosis. Failure to quickly identify the diagnosis as TPP can be fatal, but efficient correction of potassium as well as the hyperthyroid state can resolve symptoms rapidly and prevent complications. Furthermore the discovery of a mutation in KCNJ18 gene may well provide us with greater insight into the pathophysiology of thyrotoxic periodic paralysis with a view to better managing the condition.

## Figures and Tables

**Table 1:** Triggers of periodic paralysis attacks.

Triggers	Examples	Notes
High glycaemic index foods	Sweetened drinks, pasta, certain fruits, white bread, certain cereals and fruits, for example, watermelon, rice, potatoes	High glycaemic index foods promote release higher insulin levels

Salt/high sodium intake	High-salt-containing foods	Promotes diuresis and loss of potassium

Stresses	Infection, psychological, surgery	Release of catecholamines which can activate Na/K/ATPase pump

Ambient temperature	Cold weather	

Physical activity	Rest after significant exertion	Weakness maybe apparent first thing in the morning

Gastrointestinal	Diarrhoea	Loss of potassium

Drugs	Acetazolamide, oestrogen, diuretics, laxatives, liquorice, cortisol, aminoglycosides, acrolides, fluoroquinolones	Listed antibiotics adversely affect neuromuscular transmission
		Liquorice and cortisol promote potassium excretion
		Oestrogen can increase insulin resistance
